# Evaluation of Inconel 718 Metallic Powder to Optimize the Reuse of Powder and to Improve the Performance and Sustainability of the Laser Powder Bed Fusion (LPBF) Process

**DOI:** 10.3390/ma14061538

**Published:** 2021-03-21

**Authors:** Konrad Gruber, Irina Smolina, Marcin Kasprowicz, Tomasz Kurzynowski

**Affiliations:** 1 Center for Advanced Manufacturing Technologies (CAMT-FPC), Faculty of Mechanical Engineering, Wroclaw University of Science and Technology, ul. Łukasiewicza 5, 50-371 Wroclaw, Poland; iryna.smolina@pwr.edu.pl (I.S.); tomasz.kurzynowski@pwr.edu.pl (T.K.); 2 Faculty of Mechanical Engineering, Wroclaw University of Science and Technology, ul. Łukasiewicza 5, 50-371 Wroclaw, Poland; marcin.kaspro1@gmail.com

**Keywords:** Inconel 718, powder recycling, powder reuse, laser powder bed fusion, selective laser melting, additive manufacturing

## Abstract

In this paper, a detailed assessment of Inconel 718 powder, with varying degrees of degradation due to repeated use in the Laser Powder Bed Fusion (LPBF) process, has been undertaken. Four states of IN718 powder (virgin, used, overflow and spatter) were characterized in terms of their morphology, flowability and physico-chemical properties. Studies showed that used and overflow powders were almost identical. The fine particle-size distribution of the virgin powder, in which 50% of particles were found to be below the nominal particle-size distribution (PSD), was recognized as the main reason for its lower flowability and the main cause of the differentiation between virgin, used and overflow powders. Only spatter powder was found to be degraded enough to preclude its direct LPBF reuse. The oxygen content in the spatter powder exceeded the limit value for IN718 by 290 ppm, and aluminum oxide spots were found on the spatter particles surfaces. Laser absorption analysis showed 10 pp higher laser absorption compared to the other powders. The results of evaluation showed that IN718 powder is resistant to multiple uses in the LPBF process. Due to the low degradation rate of IN718 powder, overflow powder can be re-enabled for multiple uses with a proper recycling strategy.

## 1. Introduction

Additive manufacturing (AM) involves layer-by-layer manufacturing methods and has become more and more popular in the last decade. According to the ISO/ASTM 52900:2015 standard, AM has the following seven process categories: Binder Jetting (BJ), Directed Energy Deposition (DED), Material Extrusion (ME), Material Jetting (MJ), Powder Bed Fusion (PBF), Sheet Lamination (SL) and VAT Photopolymerization (VAT). Within those seven categories, more than half use powder as an input material: BJ, DED, MJ and PBF. Currently, out of all those powder-based technologies, the most popular is PBF, which has already been implemented in, among others, the medicine, aviation and automotive industries [1]. Additive Manufacturing and Laser Powder Bed Fusion (LPBF) may not be the most energy-efficient process [2], but with regard to the whole value chain around AM/LPBF, it has become highly efficient, especially in terms of material efficiency (low waste) and post-processing. However, the benefits of using the LPBF process became evident in the aerospace industry, when the “buy-to-fly” ratio, taking into account and using AM technologies, led to a reduction in the waste of material and a decrease in the amount of energy used [3,4].

One of the fundamental principles of the industrial implementation of additive manufacturing is the ability to maintain the repeatability of the process [5]. The critical aspect of this repeatability is ensuring the stability of the properties of the input materials. From the ecological and economical perspectives, powder quality and recycling are a key aspect of PBF sustainability and performance.

Depending on the processed material (metal, polymer) and the energy source used for its processing (heater, laser, electron beam), PBF is characterized by different material recycling rates. Polymer-based PBF, due to its thermal degradation, has a low level of powder recyclability. The powder that is not used to produce parts should be mixed before each process using a system- and material-specific virgin-to-used powder ratio [6]. Metal PBF, however, has a high powder recyclability, as metals are characterized by a slower degradation during the PBF process [7,8,9]. Nevertheless, the changes that occur in metal powders during their multiple reuses in the LPBF process should not be underestimated. Even small differences between the powders used to produce parts, e.g., chemical composition and particle-size distribution (PSD), can lead to significant differences in the final products, e.g., on the density of materials and their strength [10,11,12]. The possibilities for the analysis of powder materials are immense. However, the research problem involves linking the properties of powders and their changes during multiple uses with the properties of manufactured parts, as well as the ability to appropriately detect subtle differences between the powders. This finding will ultimately allow the methodology of testing and recycling powders during their multiple uses to be defined.

It is assumed that the metal powders used in LPBF are nominally spherical with a particle-size distribution that is optimized for both (1) the bulk density to fit adequately within the layer and (2) the layer thickness used. This is due to the fact that the particles cannot be too small or too large for the layer thickness [13]. Other essential features of powders also include their morphology, the resulting flowability, and several physicochemical properties, such as chemical composition, laser absorption or humidity [9,14,15]. The methods of evaluating additive materials used in additive technologies are most often borrowed from other fields of knowledge, e.g., powder metallurgy or pharmacy, and are very often standardized. These methods are adapted to the needs of additive manufacturing, and attempts are made to correlate the properties of the powders with the final effects of processing. There is no simple translation of the measured parameters into the finished product. The influence of the parameters and the differences between powder batches depends on the type of material used, its processing parameters, or the design of powder feeding systems implemented in PBF machines.

In recent years, more and more attempts have been made to determine the relationship between the measurable properties of powders and the effects of AM processes (as well as the stability and repeatability of the AM processes). However, due to the multitude of measurement parameters, materials used and other factors influencing the result, the literature is still far from formulating universal conclusions. The works in the AM powder investigation could be divided into three areas, the first of which concerns the control of powder supplies, the second the topic of powder recycling, and the third multidimensional considerations.

Several papers (Riener et al. [10] and Geisert et al. [16]) stated that the best repeatability and the highest real density of samples produced from various powders were obtained for a spherical powder that was characterized by a particle-size distribution of 10–45 µm. According to research carried out by Sudbrack et al. [11], the variable content of N in powders from different suppliers (argon or nitrogen atomized) may affect the recrystallization rate of the LPBF-ed alloy during heat treatment and the morphology of carbides and nitrides, in turn affecting the mechanical properties of the final parts. Compared to powders with a low N content, specimens produced from powders with N > 500–600 ppm showed slow recrystallization due to particle pinning of the grain boundaries by highly stable minor phases. This effect also applies less to C, which forms carbides (Nb,Ti)C.

Cordova et al. [17] examined several different powders (Inconel 718, Ti6Al4V, AlSi10Mg and Scalmalloy) after their repeated use in L-PBF technology and found that flow properties of powder increase with multiple uses. Inconel 718 powder was found to be the most resistant to property degradation. Grainger [9] tested Ti6Al4V ELI (Grade 23) powder subjected to 38 uses. A slight increase in the content of oxygen after 25–30 use cycles (approaching the normalized limit for the Ti6Al4V ELI Grade 23 alloy) and an increase in the particle-size distribution and powder flowability were detected. There were no changes in the tensile strength of the witnesses’ samples. On this basis, the conclusion can be drawn that the powder can be processed in LPBF multiple times. However, the level of its impurities should be monitored, and it is recommended to mix the used powder with the virgin one in order to maintain the appropriate particle-size distribution. Ardila et al. [18] presented results regarding Inconel 718 powder used 7-times and 14-times. As a result, apart from the change in the PSD, the impact of powder multiple use on the change of microstructure, the chemical composition, the porosity, and the static strength of the witness samples produced from those powders was not measured. However, the authors note that the low accuracy of performed measurements was not enough to finally make a conclusion on the influence of powder reuse on the properties of Inconel 718 LPBF parts. The authors of [19] emphasize that the reusability of Inconel 718, even though it is chemically stable, could be restricted due to the deterioration of physical properties like flowability and morphology.

Kiani et al. [20] proved that the density of a powder alloy had the most significant impact on powder flow properties. The less dense the powders alloy, the more prone the powder is to flowability changes. Snow et al. [21] compared the results from the novel test stand, simulating the operation of an LPBF recoater, with other popular measurement methods of powder flow. They concluded that the best correlation between simulated layer deposition and measurements results were found for the angle of repose (AOR) parameter, obtained with a rotating drum analyzer. Spierings et al. [22], based on the research of 14 iron powders and 9 nickel powders, formulated the justification for the use of the dynamic flow measurement method (rotating drum) for determining the flow limit of powders for additive manufacturing. Moreover, they proposed an attempt to standardize the method in the ASTM and ISO standards.

The presented literature analysis showed that the undertaken topic of powder reuse, recycling, and quality control is essential to the LPBF process’s performance. Even minor differences between the powders used in LPBF can result in significant differences in production batches of the finished products. As Inconel 718 is mainly an aerospace material, powder handling and reuse capabilities need to be linked with its attributes, such as chemistry, morphology and flowability, to allow LPBF process qualification and certification, and to develop effective feedstock quality control methods [23]. In connection with the above, the undertaken research responds to this demand. The research problem, however, is to link the changing properties of powders with the appropriate detection methods and respond to these changes. Therefore, in this paper, a detailed assessment of Inconel 718 powder, with varying degrees of degradation due to repeated use, was undertaken. Four states of the same Inconel 718 powder (virgin, used, overflow and spatter) were characterized in terms of their morphology, flowability and physicochemical properties. An extensive evaluation of the powders was performed, and conclusions were identified on the IN718 powder degradation and its recycling performance during repeated use in the LPBF production cycle, as well as on the effectiveness of the measurement methods used for IN718 powder evaluation.

## 2. Materials and Methods

### 2.1. Powders Used in the Study

Inconel 718 powder purchased from SLM Solutions Group AG, a German manufacturer of LPBF machines, was used in the study. The powder is certified according to the EN 10204:2004 standard. The chemical composition of the powder, declared by the powder supplier, is presented in Table 1. The study used powder from a single production batch, with a total weight of 150 kg. The powder was used in the LPBF production cycle, which lasted for 12 months and consisted of 20 full-powder reuse cycles. The LPBF processes were carried out using the SLM 280 2.0 Dual machine (equipped with a 1070 nm fiber laser with a max. power of 700 W) under a protective atmosphere of technical argon (O_2_ level below 100 ppm, purity class 4.6).

Four states of the described powder, i.e., virgin powder (V), powder after multiple uses (U), overflow powder (O) and spatter powder (S), were studied. The types of powder states used in the research are described in detail in Table 2.

It is worth mentioning that in regular industrial practice, overflow and spatter powders are treated as waste. Both are rejected during successive screening cycles in between LPBF processes and are not used further for parts production in an industrial LPBF production process. From the initial 150 kg of the virgin powder, ≈50 kg of used and ≈15 kg of overflow powders remained. Moreover, during the last three LPBF processing cycles, ≈0.5 kg of spatter was collected. The rest of the powder was used to manufacture parts (≈75 kg) and was filtered out during the process/machine cleaning after the LPBF process (≈10 kg).

Before the measurements, each of the powders was heated in an industrial furnace (at the temperature of 70 °C for 24 h) to eliminate the possible influence of moisture on the results. Between testing, the powder was stored in airtight, moisture-proof containers with moisture-absorbing silica gel packets inside. All the measurements were performed in a room with controlled conditions, i.e., at an ambient temperature of 21 °C, 50% relative humidity and a pressure of 1013 hPa in order to eliminate the influence of environmental conditions on the flow properties and flowability [27].

### 2.2. Research Methods

To investigate the differences between the described Inconel 718 powder states, the most important properties of metallic powders, in terms of their use in LPBF, were selected and measured according to standardized and non-standardized procedures (Table 3). Powder properties were divided into three sections, i.e., powder morphology, flow properties/flowability and physico-chemical properties. The detailed conditions of the performed measurements are presented in accordance with the presented classification.

#### 2.2.1. Powder Morphology

The laser diffraction system used in this study consisted of a HELOS BR R4 + RODOS laser diffractometer and a VIBRI dispersion unit (Sympatec GmbH, Clausthal-Zellerfeld Germany). The measurement parameters used are presented in Table 4.

Statistical analysis of the results was carried out automatically using PAQXOS 3.1 software (Sympatec GmbH, Clausthal-Zellerfeld, Germany) according to the Fraunhofer procedure (acc. to ISO 13320:2009). To enable comparison of the data with image analysis, the PSD area parameters, i.e., the fractional area distribution density q_2.LD_(x_m_) and cumulative area distribution 1-Q_2.LD_(x_i_), were calculated. For the particle-size distribution function q_2_ (x_m_), the following parameters were also determined: standard deviation (σ), skewness (central moment M_3_) and kurtosis (central moment M_4_).

SEM images were obtained using an EVO MA25 microscope (Carl Zeiss AG, Oberkochen, Germany) equipped with an EDS detector. The powder’s morphology and shape, and the surface condition of the individual particles, were characterized. The SEM images were also used to calculate the particle-size distribution and the powder particle shape factors. Analysis was performed based on binarized images and ImageJ 1.53a software (Wayne Rasband from National Institutes of Health and Laboratory for Optical and Computational Instrumentation (LOCI, University of Wisconsin), USA). For each particle, its equivalent projected area was determined, and its dimension was reduced to the equivalent projected area diameter. The particles were then assigned to the 31 measurement classes, the same as the laser diffraction measurement classes, in order to ensure comparability. The fractional area density distribution q_2.IA_(x_m_) and cumulative area distribution 1-Q_2.IA_(x_i_) were determined.

The aspect ratio was expressed as the ratio of the most extensive diagonal measured to the smallest diagonal of the particle measured. Circularity was determined by ISO9276-6 according to formula (1):

(1) $$C=\sqrt{\left(\frac{4\pi A}{P^{2}}\right)}$$

where *A*—the surface of the powder particle; *P*—particle circumference.

#### 2.2.2. Flow Properties and Flowability

The powders were analyzed to determine the flow properties and flowability with the use of general, standardized powder measurement methods:

- Flowability analysis using a Hall funnel (ISO 4497, ASTM B213-20). The measurement was performed with a Flow Meter SLM-powder funnel (SLM Solutions GmbH, Lübeck, Germany) with a non-standard measuring opening of 3.81 mm (0.15″), a standard α = 30° cone angle and a manual stopwatch.
- Bulk density (ISO 3923-1, ISO 3923-2, ISO 3923, ASTM B329) was measured using an EV2 volumeter (Electrolab, Mumbai, India) and an XS2002S laboratory balance (Mettler-Toledo, Columbus, OH, USA).
- Tapped density (ISO 3953, ASTM B327) was measured using an ETD 1020 automatic tester (Electrolab, Bombay, India), with 100 mL of powder carefully being poured through the Hall funnel into the cylinder of the analyzer. Five hundred strokes were then performed in an automated cycle at a maximum frequency of 100 Hz.
- Angle of Repose (AOR) was determined using the Hall funnel BEP2 Flowability Tester Upright & Stand (Copley Scientific, Nottingham, UK) and the Digimatic HDS Absolute altimeter (Mitutoyo, Kawasaki, Japan).
- The Hausner coefficient was calculated as the ratio of the free bulk density to the tamped density.

The method, which in line with the literature [22,29,30] more accurately simulates the dynamic nature of the flowing powder during the LPBF process, involves analysis with the use of a rotating drum. Each of the powders was characterized based on a sample with a standardized volume of 55 cm^3^ [31]. The measurements were performed with a GranuDrum material flow analyzer (Granutools, Awans, Belgium) in the hysteresis mode. Cohesion index, avalanche angle α_AA_ and first avalanche angle α_fAA_ were calculated. The principles of measurements can be found in [32]. The measurement parameters used in the study are summarized in Table 5.

#### 2.2.3. Physico-Chemical Properties

To detect subtle changes in the chemical composition of powders, three representatives of alloying elements (Cr, Al, C) and three impurity elements (H, N, O) were measured for each powder with the use of spectrometry and high-temperature extraction methods:

- Al, Cr—by the OES ICP method on the 5100 ICP-OES system (Agilent Scientific Instruments Ltd., Santa Clara, CA, USA).
- C—by the HFIR method on the CS-600 system (Leco Corp., St. Joseph, MI, USA).
- H, N, O—high-temperature extraction method on the TCHEN 600 system (Leco Corp., St. Joseph, MI, USA).

Phase analysis was performed using a MiniFlex 600 X-ray powder diffractometer (Rigaku Corporation, Tokyo, Japan) with a 2θ geometry ranging from 30° to 100°. The measurement was performed with a 0.001° leap. The hardness of the powders was measured with the ZHVμ-A microhardness tester (Zwick-Roell Group, Ulm, Germany).

To verify the presence of internal defects, cross-sections of the powder particles were also analyzed. For these tests, the metallographic samples (a mixture of powder and metallographic resin) were prepared, ground with SiC sandpaper and then polished using a suspension of SiO_2_ nanoparticles.

For the hardness measurements, metallographic samples of the powders from the microscopic examination were used. The hardness was measured for each batch of powders, with only large particles (dia. > 40 µm) selected in order to obtain the appropriate size of the measuring surface. Vickers hardness profiles on the cross-sections were determined at 0.98 N. For each sample, 10 indents were performed.

Analysis of the laser absorption of the powders was performed using the Exemplar Plus Spectrophotometer (model BTC655N-ST1) and BIP2.0 Integrating Sphere equipped with a 20W Tungsten Light Source (B&W Tek, Newark, DE, USA). The system allows measurements of the laser absorption of materials with the possibility of modulating monochromatic light in the range of 900–1100 nm.

#### 2.2.4. Porosity of the LPBF-Ed Control Specimens

Porosity levels of the LPBF-ed control specimens (dim. 10 × 10 × 10 mm^3^), produced from V and U powders, were assessed by computer image analysis. Standard process parameters for IN718, provided by the manufacturer of the LPBF system, were used. Five samples were tested per each powder state. Polished cross-sections were prepared on the planes parallel to the specimens’ building direction (z-axis of the LPBF system). Images obtained using a confocal laser scanning microscope OLS 4000 LEXT (Olympus Corp., Tokyo, Japan) were subjected to binarization. Porosity was determined as the percentage of black pixels concerning the total number of pixels of each individual cross-section image.

## 3. Results and Discussion

### 3.1. Powder Morphology

**Figure 1:**
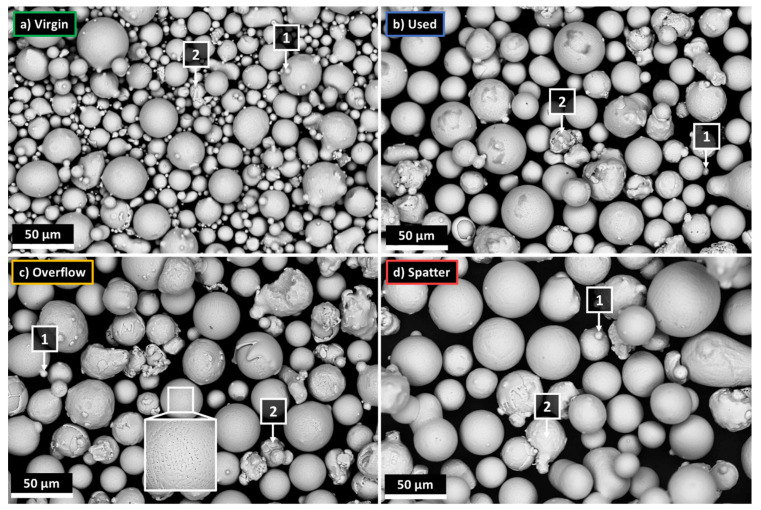
Microscopic images of the IN718 powders in various processing states, SEM/BSE; (**a**) virgin (V); (**b**) used (U); (**c**) overflow (O); (**d**) spatter (S).

Due to the small sample of the powder collected for microscopic examination in relation to its entire population, the characteristics of the powders may depend on the place of their collection. Therefore, the powders were collected and analyzed several times for each of the powder states. Figure 1 presents SEM images of the representative samples of each of the four tested powders.

On this basis, the following visual differences between the powders can be identified:

- The virgin powder has the highest proportion of small particles (less than 10 µm) when compared to the other powders, as well as the highest number of satellites (1).
- The number of satellites (1) is the smallest for the spatter powder, and comparable in the case of the overflow and used powders.
- There is no difference between the overflow and used powders that can be detected comparing SEM images alone.
- The spatter powder contains the highest number of large particles and particles exceeding 45 µm in diameter, which suggests that the spatter particles generated during the LPBF process are larger than the particles of the virgin powder, as the nominal PSD is between 10–45 µm.
- Most of the powder particles, regardless of the powder’s state, are characterized by a spherical or spheroidal shape.
- All the powders contain some proportion of non-spherical particles with a highly developed surface. They are most often single particles or groups of particles connected by solidified material (2). They could arise as defects during the gas atomization process (2) due to the collision of already formed particles with non-solidified or partially solidified material [33]. The number of such particles, as shown by microscopic images, increases with the successive cycles of using the powder. Such particles can also be formed in the LPBF process as a type of spatter, in this case by collisions between metallic jet spatter and solid spatter [25].

**Figure 2:**
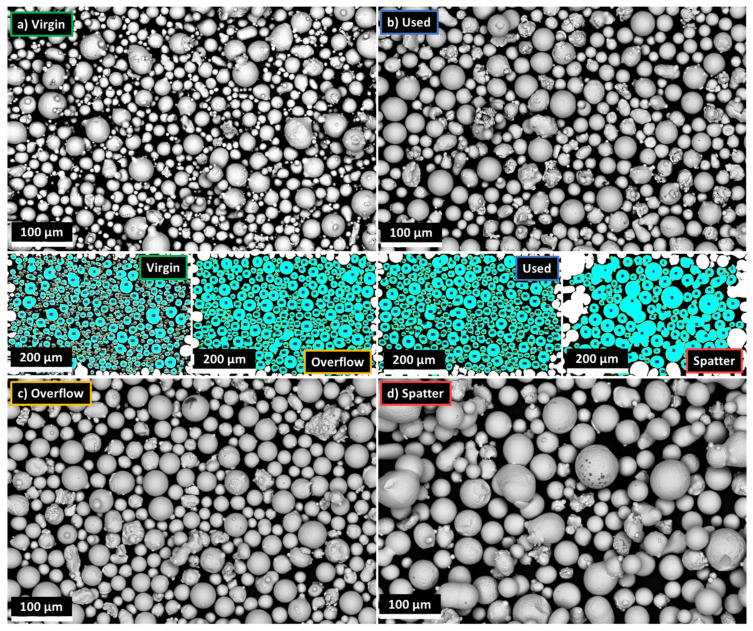
Microscopic images of the IN718 powders in various processing states, SEM/BSE and binarized images with a counting mask applied; (**a**) virgin (V); (**b**) used (U); (**c**) overflow (O); (**d**) spatter (S).

Figure 2 shows a sample image analysis that was performed to determine the particle-size distribution and particle shape factors. A mask was applied to the binarized images, which present the individual counted particles (area—aquamarine, outline—yellow). Measurement data, calculated based on the presented particles’ masked images, were compared with the particle-size distributions obtained by laser diffraction.

**Figure 3:**
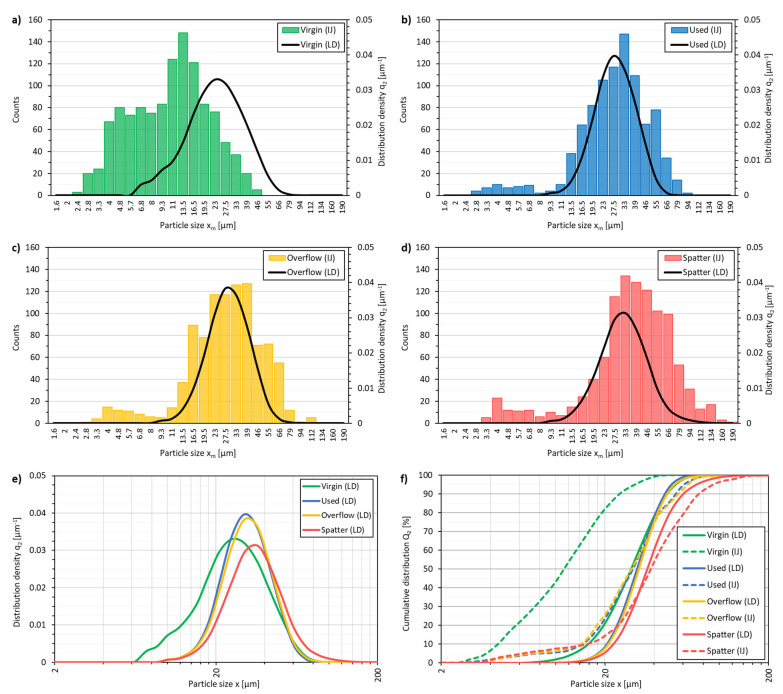
Comparison of the obtained particle size distributions of the IN718 powders in different states; measurement results using laser diffraction (LD) and image analysis (IJ); (**a**) virgin (V); (**b**) used (U); (**c**) overflow (O); (**d**) spatter (S); (**e**) distribution density comparison; (**f**) cumulative distribution comparision.

Figure 3a–d presents the comparison of the density distribution of q_2.LD_(x_m_) obtained from laser diffraction with the histograms of q_2.IA_(x_m_) from the SEM image analysis for each powder state separately. Figure 3e presents a comparison of the density distribution of q_2.LD_(x_m_) for all the powder states, and Figure 3f shows both the 1-Q_2.LD_(x_i_) and 1-Q_2.IA_(x_i_) plots. With the virgin powder, the shift between the image analysis and the diffraction is very large.

The distribution obtained for the analyzed powder images in the V state is characterized by the median particle equivalent of x_50.2_ = 11.25 µm, and in the case of laser diffraction, x_50.2_ = 34.60 µm (Table 6). For the S powder, the median for the image analysis is also lower (x_50.2_ = 37.71 µm, and x_50.2_ = 42.15 µm) than for diffraction. However, the difference is much smaller (Figure 3a,d), and the x_90.2_ obtained from both methods is equal. The U and O powders show similar results, with x_10.2_ and x_50.2_ being smaller, and x_90.2_ being higher for the image analyzed PSD. Despite the small differences, the results for the U and O powders are the most comparable for the laser diffraction and image analyzed PSDs. Despite the differences observed in the particle size distributions, the shapes of the laser diffraction distribution curves follow the image-analyzed PSD histograms. The V powder shows many small particles when compared to the rest of the powders. The S powder is characterized by a very large number of large particles, which is beyond the nominal PSD of the virgin powder. This trend applies to both the laser diffraction and image analysis data. It can also be seen that the laser diffraction data detects the presence of very small particles (<5 µm), despite their presence in all the powder batches.

The divergence of the results is caused by the method of determining the equivalent projected area between both measurement methods. In the case of SEM measurements, the contrast that is set during microscopic observations is crucial in order to obtain sharp particle edges for binarization. During binarization, a consensus must also be found between the precise determination of the particle edges and for when the particles are in contact. Connected particles can be considered as one collapsed particle. To avoid this, the binarization threshold towards the center of the powder particles is increased, in turn reducing the area of the particle image. At the same time, laser diffraction also has its drawbacks. The standard optical model for light scattering of particles, i.e., Fraunhofer diffraction theory, does not require the knowledge of the refractive index of the particles. For this reason, it will be inaccurate in the case of measurements of very small particles, where a better solution is a model based on Mie theory [34]. This model is more demanding in terms of application because it requires additional optical information about the tested particles. All these factors mean that the PSDs determined by the two used methods cannot be directly compared.

Nevertheless, the trends are maintained, and both methods can be used interchangeably, depending on the available equipment. Data obtained by both methods show large differences in the PSD of the V and S powders compared to the other powders, and very small differences in the PSD of the U and O powders in relation to each other. Based on the PSD results (Figure 3e–f), a hypothesis regarding the tendency of powders to eliminate the fine particles during subsequent cycles of powder use and powder recycling between LPBF build jobs can be drawn. The difference in the PSD between the V, U and O powders is meaningful when considering small particles (below the dia. of 15 µm). The smaller particles melt and sinter easier, forming satellites near the melting areas [17]. The fine powder particles are also the lightest, which leads to floating in the form of metal dust and its deposition on many of the surfaces, in the chamber of the LPBF device, in the screening device, and within the powder transportation and process gas and filter systems of both the LPBF and peripheral devices. In the scope of larger particles, the virgin, used and overflow powders are characterized by a similar x_90.2_ (between 50-56 µm, regardless of the powder state and measurement method).

The function parameters of q_2.LD_(x_m_), i.e., standard deviation (σ), skewness (M_3_), kurtosis (M_4_) and Span, describes the shape of the PSD curves. The skewness and kurtosis of the normal distribution are 0. Therefore, the distributions nearest to normal are seen in the U and V powders. Concerning the normal distribution, they are slightly right-skewed and slightly more concentrated around the mean value. However, the used powder has a narrower PSD, as evidenced by the lowest Span and σ parameters, which is mainly due to the loss of small particles. The overflow and spatter powders show a tendency for a right-skewed PSD, which proves that the distributions are shifting towards larger particles. Note the significant difference between the skewness (0.64 to 0.94) and kurtosis (0.45 to 2.03) parameters for the used and overflow powders. These are the only analyzed PSD parameters, based on which it is possible to determine clear differences between the powders.

The shape of the analyzed particles was defined as spherical based on the measured AR and circularity coefficients. A small amount of irregularly shaped particles increases the mean AR and lowers the circularity. Compared to similar powders, all of the analyzed powders are characterized by good sphericity [17]. The used and overflow powders have coefficients that are the closest to 1 (1 = circle/sphere), while the virgin powder is the most distorted, followed closely by the spatter powder.

Each of the powders contains a large common part, i.e., particles that have not changed morphologically because they have not been melted or distorted during the LPBF process. It should be remembered that it is the same powder in different states. From a PSD point of view, overflow powder morphology allows for its reuse. The used industrial powder screening device is set to be fast. This causes a large amount of overflow powder to be collected, and in the case of this study, it is ≈5–10% of the initial powder batch, which is treated as waste. In terms of morphology, it is almost the same powder as the reconditioned and ready-to-use used powder.

### 3.2. Flow Properties and Flowability

Table 7 presents the summarized results of the flow properties analysis, i.e., bulk and tap density, Hausner’s coefficient and Hall funnel flow time. The analyzed bulk density, tap density and Hausner coefficient for the U, O and S powders are identical within the measured deviation, expressed as the standard deviation. The only significant difference in flow properties is detected between the virgin powder and the other powders. The virgin powder is characterized by a lower bulk density and tap density, and at the same time a higher packing density, as evidenced by the higher Hausner coefficient. If the descriptive classification scale of flow based on the Hausner coefficient was assigned to the powders, all the materials, except the V powder, would be characterized as “Excellent” [35]. The value of the Hausner coefficient for the virgin powder (1.13 ± 0.01) classifies it as “Good”. Such a difference is expected and is related to the broader PSD of the V powder and the high proportion of fine particles, which in turn deteriorate the flow properties. The U, O and S powders are characterized by a more homogeneous particle-size distribution (without the smallest particles). This translates into better flow property values. Nevertheless, the difference between the powders is small, as the “Good” level starts at values ≥1.12.

**Figure 4:**
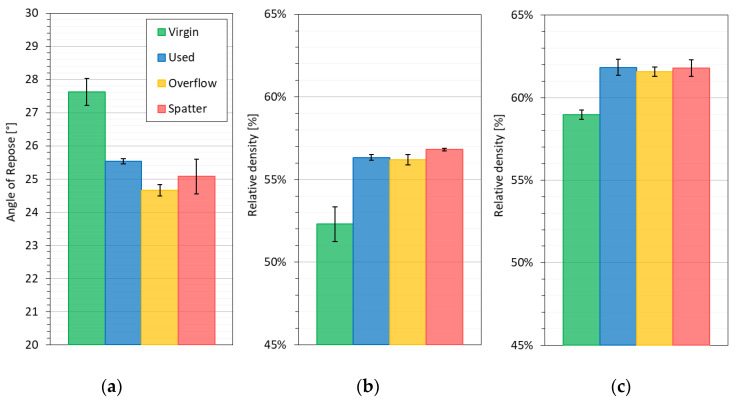
Parameters of the static flow properties of the powders with regard to the sample series (V, U, O, S): (**a**) Graph of the angle of repose (°); (**b**,**c**) Comparison of the bulk density and compacted density of the powders expressed as the % density of the IN718 alloy (8.19 g/cm^3^).

The descriptive classification scale is also used to measure the angle of repose. The R.L. Carr index, which is based on AOR, is used to describe the flow properties of powder materials [36]. According to this classification, all the powder samples showed the “Excellent” level (AOR < 30°) [37]. In some cases, Carr’s index is also used to determine the compressibility of the powders [27]. In the case of AOR analysis, small differences between the powders appeared. The highest flowability was obtained for the O powder, followed by the U powder (Figure 4a). The S powder is characterized by a high standard deviation, and therefore the differences between the S, O and U powders cannot be determined. The virgin powder again showed good but, at the same time, significantly lower flowability than the other powders.

The longest flow time was measured for the virgin powder, followed by the spatter powder. The lowest flow time was obtained for the overflow and used powders. All the powders flowed without funnel jamming. The higher flow time should be attributed to the PSD of the S and V powders. Due to the non-standard hole diameter (3.81 mm/0.15″) of the Flow Meter SLM-powder device, it is not possible to directly compare the results with the literature values for Hall (2.54 mm/0.10″) or Carney (5.08 mm/0.2″) Funnels. The flow time determined for the V powder is slightly lower (6.04 ± 0.16 s) than the value declared for the entire batch by the manufacturer’s certificate (6.25 s). The difference, however, is minimal and may result from differences in the response time of the stopwatch operator.

The typical and correct layer density in powder-based additive manufacturing is set in the literature in the range from 40% to 60% of the powder’s material real density [38] (Inconel real density is 8.19 g/cm^3^). Figure 4b,c shows the relative density of the powders concerning the density of the solid material, and the relative density of the powders, expressed as bulk and tap density. The relative bulk density of the U, O and S powders is over 56%, and for the V powders, it is about 52% (4pp difference). The difference drops to 3pp for the powders after tapping (Figure 4c). However, the measured difference is high enough to affect the porosity level of objects produced using LPBF. It has been proven many times [10,12,39] that it is possible to obtain low porosity from powders with different flowability parameters and a different PSD. Nevertheless, to maintain porosity at a low level, the LPBF’s process parameters (e.g., laser power, scanning speed or hatch distance) need to be individually adjusted when the powder properties are different.

**Figure 5:**
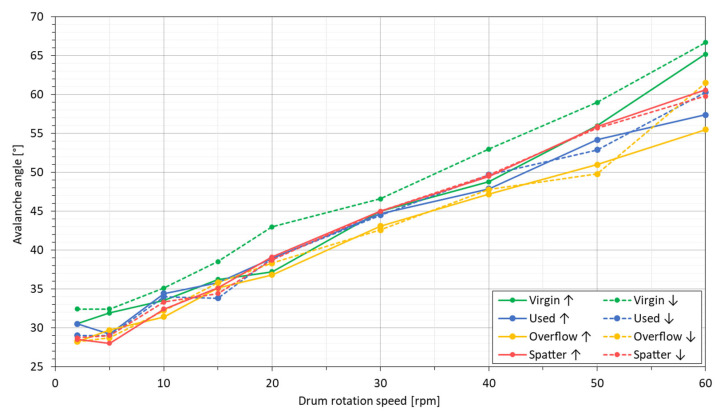
Avalanche angle diagram for the IN718 powders in various processing states with regard to the rotational speed of the measuring drum.

According to review paper [10], bulk density and tap density cannot be directly compared with the density of the layer applied during the LPBF process, as these powders’ characteristics do not reflect the dynamic interactions between powder particles during the application of layers in the powder bed processes. Therefore, it is becoming more and more popular to analyze powders in terms of flowability with novel methods, such as measuring the cohesion index and avalanche angles with the use of rotating drum analyzers. In Figure 5, an avalanche angle (α_A_), as a function of drum ration speed for all the investigated powders, is shown. The solid lines show the results for an increasing drum rotation speed (↑), and the dashed lines show the results for a decreasing drum rotation speed (↓). The avalanche angle increases almost linearly for all of the powders. Only the virgin powder shows a slight difference at a decreasing rotation speed.

A linear regression y = ax + b for all of the curves reduces the graphs to the form of single values. By determining the average slope “a” of the linear regression y = ax + b for the two curves, the information concerning the slope angle of the curves a = tg(α) was obtained (Table 8). The virgin and spatter powders have a greater slope of the linear regression curve, i.e., their flowability, understood here as the avalanche angle, is more dependent on the rotation speed than for the other U and O powders.

**Figure 6:**
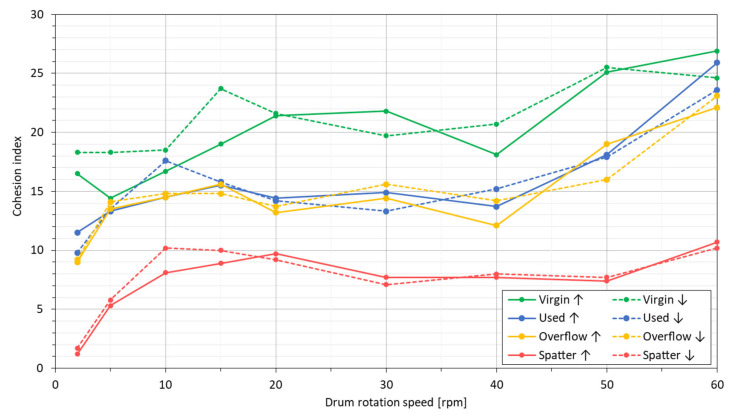
The cohesion index for the IN718 powder in different processing states with regard to the rotational speed of the measuring drum.

The dynamic nature of the powder measurements allows the simultaneous influence of the four major mechanisms that affect powder flowability to be presented. These include (1) friction between particles, (2) mechanical interlocking, (3) interparticle forces such as Van der Waals, and (4) liquid bridging [39,40]. By comparing the flow properties determined with the use of static or semi-dynamic methods with the flowability expressed by the dynamic flow properties (i.e., the cohesion index), essential differences in the flowability between the powders, which were not registered before, were detected. A clear difference between the flowability of the U and O powders in comparison to the S powder is visible (Figure 6). During the traditional flow properties analysis, all three powders, despite large differences in PSD, showed almost the same flow properties. The presented correlation between individual powder states is consistent with literature findings, where powders with a higher PSD are characterized by better layer spreadability in the powder bed process itself [7,41,42].

The authors of work [43] observed a strong relationship between the spreadability and the cohesion index, as well as the influence of the recoater speed on the spreadability of the powders. It is possible to estimate the optimal speed of the recoater distributing the powder layer in the LPBF process. For the 55 mm diameter measuring drum used in this study, the rotation speed of 10 rpm corresponds to a recoater speed of ≈60 mm/s. In the case of the tested powders, the flowability is stable in the range from 20 to 40 rpm for the V, U and O powders, which corresponds to a recoater linear speed of 120–240 mm/s. This translates well into the recoater speed in the performed LPBF processes, in which a recoater speed of 200 mm/s was used. Moreover, no errors in layer deposition were observed. It can be noticed, for the S powder, that stable properties remain with a drum rotation speed from 30 to 50 rpm (180–320 mm/s recoater speed respectively). At a low rotation speed (>5 rpm), the flowability of the used, overflow and spatter powders improves, while at a high rotation speed (>50 rpm), the cohesion index increases significantly for all the powders. The average cohesion index (Table 8) was calculated in order to obtain a single value property for further analysis. On the descriptive flowability scale presented in [31], the S powder is categorized as “Good”, the used and overflow powders as “Fair”, and the virgin powder as “Passable”. The first avalanche angle αAA confirms the obtained classification, but the difference for the used and overflow powders is slightly more significant than when measured with the cohesion index.

From the perspective of the LPBF process, the flowability of overflow powder allows for the O powder to be reused. The obtained flowability and layer spreadability are very similar to the used powder. In this respect, the virgin powder fares worse, especially when the cohesion index is considered, as it only classifies the powder as “Passable”. It should be emphasized that the comparable flowability of the used and overflow powders is very important due to the possibility of mixing the powders and applying the same LPBF process parameters to both powders. What is more, the repeatability of the production cycle can be maintained due to the fact that the powder has multiple uses. At the same time, the use of only virgin powder, due to its lower flowability, may affect the obtained results of the LPBF process. It is therefore recommended, in terms of layer spreadability, to mix used powder with virgin and overflow powders in order to maintain a stable LPBF process.

### 3.3. Physico-Chemical Properties

**Figure 7:**
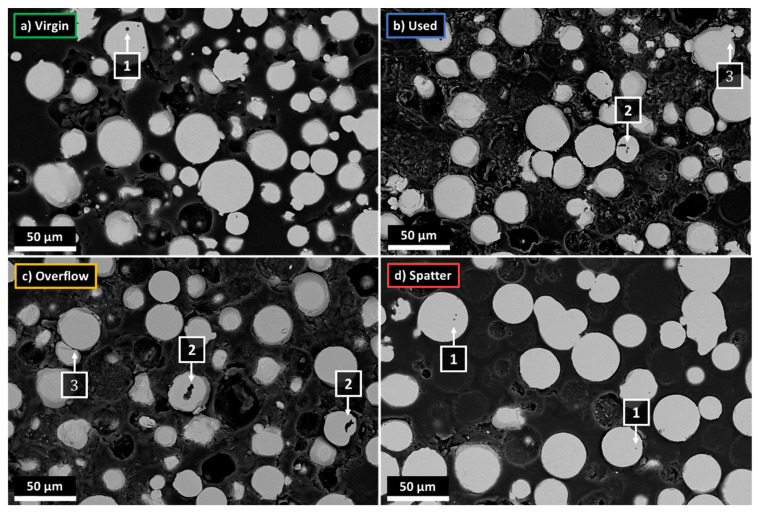
Microscopic images of metallographic cross-sections of the IN718 powders in different processing states, SEM, BSD detector: (**a**) virgin (V); (**b**) used (U); (**c**) overflow (O); (**d**) spatter (S).

Gas atomization processes can lead to the formation of gas pores (1) inside the powder particles, where two types of pores can be observed: (1) gas pores, which form when a gas particle is trapped inside the material [33], or (2) irregularly shaped LOF (lack-of-fusion) pores, when the material solidifies before the powder particles are completely formed [44]. As a consequence, particle defects can cause the porosity of the components produced from these powders by trapping the gas present in the powder inside the remelted material [18,44]. In each of the powders, a few defects in the form of both types of pores were observed, with examples shown in Figure 7. In the case of the V, U and O powders, LOF pores were found more frequently (2). Most of the pores found in the spatter sample were small (dia. below a few µm) gas-type pores (1) that were likely formed during the formation of spatter under the argon flow (gas speed was set to 22 m/s) during the LPBF process. Additionally, partially melted agglomerations of particles have cavities (3). In general, the number of internal defects is comparable with other studies [42,45,46], and should not disrupt the LPBF process, especially when the number of defects does not change within the V, U and O powders.

**Figure 8:**
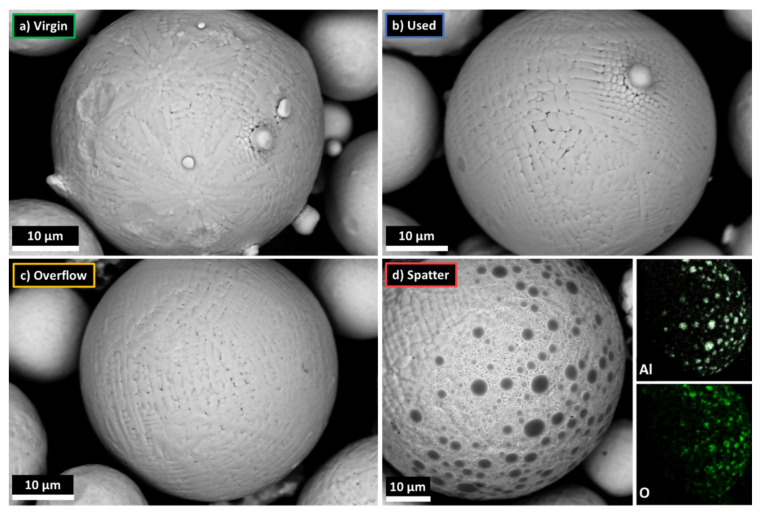
Microscopic images of the IN718 powders in various processing states, SEM/BSE; (**a**) virgin (V); (**b**) used (U); (**c**) overflow (O); (**d**) spatter (S), and EDS/SEM analysis of the area containing oxide spots.

The hardness analysis of the powder particles did not show any significant differences between all powder states (Table 9). The measured values are within the range of 240–250 HV. Powder S is characterized by a lower average hardness (by 10 HV0.1), which is a statistically insignificant difference. The measured hardness is characteristic of annealed Inconel 718 (160–320 HV) [47]. Due to the dendritic microsegregation (Figure 8) clearly visible on the spherical powder particles, which is caused by rapid cooling in the atomization process, the measured hardness is in the top half of the range. The hardness results confirm that none of the powders were exposed to high temperatures that could result in slow precipitation hardening of the material of the powder particles.

Table 10 presents the analysis of the chemical composition of all powder states. The chemical composition of powders can change along with their degradation due to contamination and may be of crucial importance for the later properties of the processed material. Six elements were selected for the analysis, i.e., Al, Cr, C, which are alloying elements, and N, O, H, which are impurities. The H content was checked, as hydrogen charging of Inconel 718 deteriorates its mechanical properties due to the hydrogen embrittlement phenomenon [48]. The oxygen content was determined due to the phenomenon of the intergranular oxygen increasing the crack growth rate that occurs for oxygen-affected and fatigue-loaded Inconel 718 [49,50]. The increase in the content of O and H will therefore mean a decrease in the properties of the material after its processing. Powders with a high N content (>600 ppm) will affect the properties of the LPBF-ed material. The recrystallization process during heat treatment will be slower due to particle pinning of the grain boundaries by highly stable minor phases, especially nitrides. This effect also applies to a lesser extent to the content of C, as it affects the precipitations of MC and M_23_C_6_ carbides [11]. Aluminum, due to its low melting temperature and high thermal conductivity, may additionally evaporate during rapid melting in the L-PBF process [51]. A lower content of Al may later affect the number of strengthening phase γ′ precipitates (Ni_3_(Al,Ti)) when the LPBF-ed material is heat-treated. The chromium content was measured for control purposes, as it is one of the main alloying elements in Inconel 718.

The measured content of the alloying elements Al, Cr and C was slightly higher than declared by the powder manufacturer, but it is within the limits of the ASTM B637 chemical composition standard. No trend was observed in the measurement results for the alloying elements, which means that small differences are most likely caused by random differences in the chemical composition between the specific samples of the powders. In the case of the impurities, both N and H showed the same content for all the samples of powders. The only negative trend was observed for O. The oxygen content increased with the degree of powder degradation during multiple uses. From an initial level of 250 ppm, the content in the used powder increased to 390 ppm, where 400 ppm is the safe limit for this contamination according to ASTM B637. The overflow powder showed a lower oxygen content than the used powder, which is probably due to the fact that it consists partly of used powder at different degradation levels (times of multiple uses), as well as spatter powder. The highest oxygen content was determined for the spatter powder (690 ppm), which suggests that the main mechanism of contamination takes place during the formation of the spatter. Despite the use of a closed process chamber in the LPBF machine and the use of shielding gas, a small amount of oxygen was still present during the LPBF process, which in turn contaminated the powder. Gasper, A. et al. [52] found spots and films of Al_2_O_3_ and TiO_2_ on the surface of the Inconel 718 spatter particles, and these are on par with the oxide spots found in this study, as confirmed by the EDS analysis (Figure 8d). The detected oxide spots are present only for the small portion of spatter particles, as can be seen in Figure 1 and Figure 2. Most of the particles are free of oxide spots. Therefore, the increase in impurities is low. As with the other reuse-dependent powder properties, the mixing of the virgin, used and overflow powder should be enough to maintain the appropriate oxygen content (O < 400 ppm) in Inconel 718 powder reused during LPBF process cycles.

**Figure 9:**
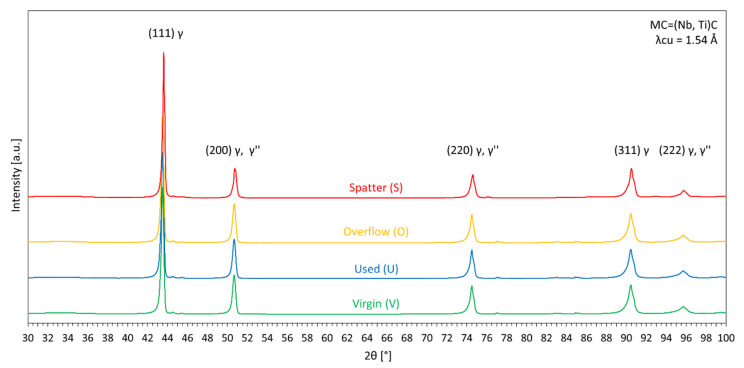
XRD spectra of the IN718 powders in all states.

Figure 9 shows the XRD diffraction spectra for each of the powders. XRD spectra measurements were performed to register possible differences in the phase composition of the powders. All the samples are characterized by the same reflections. The registered reflections for the 2θ angle correspond with the main phase of the Inconel 718—γ phase. The presence of γ′ and γ″ was excluded due to the low hardness, which is characteristic of the annealed IN718 condition. No reflections from other phases (e.g., δ, Laves, MC) were registered. This does not exclude their presence, but if they were present, they were in a very small amount. The XRD results for the powder in all conditions, as well as hardness and microscopic results discussed earlier, indicate typical microstructure of Inconel 718 powder, i.e., solid solution of γ phase with dendritic microsegregation, similar to that found in other studies [46,53]. This confirms the lack of powders’ thermal degradation, i.e., precipitation of additional phases, as all powder states are characterized by the same results. 

**Figure 10:**
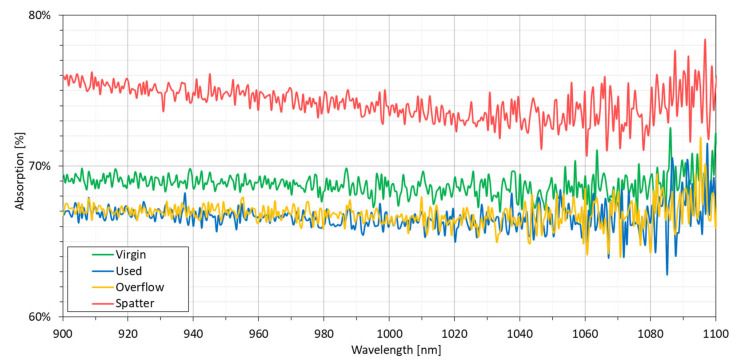
Laser absorption of the IN718 powder in all states.

Due to the fact that constant laser parameters are used in the LPBF production cycle, the absorption of laser radiation is essential in terms of the possibility of multiple powder reuses. The change in the absorbency of the powder due to different levels of material wear adversely affects the final density of the elements produced using the LPBF process [54]. The LPBF machine used in this study is equipped with a 1070 nm fiber laser, and therefore the laser absorption for this wavelength is of great importance. Due to the monochromatic light modulation method used in the apparatus adopted in this study to simulate laser light, the results are not as accurate as measurements using a real laser source with a constant wavelength [55]. Therefore, laser absorption for the entire available wavelength spectrum in the spectrophotometer apparatus was used (900 to 1100 nm) in order to allow for a more accurate detection of the differences between the powders. The results are shown in Figure 10.

The measurement results reveal both differences and similarities between the powders. The laser absorption of the used and overflow powders is the same in practice. Simulation studies showed [56], due to the local changes of powder particle sizes and the arrangement of powder particles between the individual locations in the single LPBF powder layer, that laser absorption fluctuations can be bigger than those measured between the used and overflow powders. The virgin powder, on the other hand, shows a slightly higher laser absorption, which, especially for lower wavelengths, shows a more significant difference. The average absorption (Table 11) determined for the entire wavelength spectrum is approx. 2 pp higher when compared to the used and overflow powders. The spatter powder shows a clearly visible difference and is characterized by laser absorption that is 10 pp higher when compared to the U and O powders.

Based on the morphology studies, it is known that the S powder, when compared to the other powders, is characterized by a higher proportion of large particles, a smaller number of satellites and a smaller number of the smallest particles. Experimental and simulation literature findings suggest [55,57,58] that such a PSD should show lower laser absorption due to the smaller number of particles on which laser beam reflections may occur, which is in turn due to the fact that a lower number of particles prevents multiple reflections. Multiple reflections increase the possibility of absorption because a portion of the reflected energy is absorbed after each reflection. In an experiment by Tolochko et al. [55], the absorption of high PSD powder (63–106 µm) was only slightly lower (A_1060nm_ = 0.62), as was the case for powders with a particle-size distribution below 50 µm (A_1060nm_ = 0.64). Due to the wide PSD of all the powders, the probability of reflections should be similar, and this phenomenon may be negligible.

The cause of the higher laser absorption of the spatter powder is probably due to its surface condition. Studies performed by Coste et al. [59] showed that the surface of IN625 after laser oxidation caused an increase in laser absorbance from 0.40 to 0.86. As confirmed earlier by SEM studies, aluminum oxide spots were found on some spatter particles, but the amount of spatter particles with oxide spots was low. Observation with the naked eye also revealed a difference in the color of the spatter powder, which showed a brown tilt when compared to the pure metallic grey color of the V, U and O powders. The brown tilt characterizes the surface of Inconel 718 oxidized by diffusion, as an effect of exposure to high temperatures [60]. Inconel 718 exposed to an atmosphere of technical nitrogen with low oxygen content, e.g., during heat treatment processes, is characterized by a similar surface condition. A similar atmosphere exists in the LPBF process chamber. On the other hand, SEM images of the spatter particles’ cross-section did not show a thick oxidized layer. This, however, corresponds with the oxygen measurements (an increase from 250 ppm for the Virgin powder to 690 ppm for the Spatter powder) and indicates a very thin oxidized layer. Hryha, E. et al. [61] showed, on an example of Hastelloy X nickel-based alloy powder used in the atmosphere-controlled LPBF process, that powder exposed to a laser beam is characterized by an increase in its oxide content in the form of a thin oxidized layer with a thickness of 1 to 4 nm. This confirms the hypothesis that the thin oxidized layer on the spatter powder is the main reason for its higher laser absorption.

As with the powders’ morphology and flow properties, the positive trend is maintained within the physico-chemical properties of the Inconel 718 powder in different reuse conditions. The properties of the V, U and O powders are stable, which allows for their reuse.

### 3.4. The Porosity Level and Hardness of LPBF-Ed Control Samples

To evaluate the effect of powder degradation on LPBF-ed parts, porosity level and hardness were analyzed in samples produced with use the of virgin and used powders.

The mean porosity level of 0.09 ± 0.03% was measured in the samples manufactured from virgin powder and 0.18 ± 0.04% in the samples manufactured from used powder. In both cases, the porosity level was very low, but the effect of PSD differences is slightly visible. An adjustment of the LPBF process parameters for the U powder is therefore recommended.

The hardness analysis showed no significant differences, as chemical composition and other physico-chemical properties of both powders are practically the same. The difference in the mean of the 10 measurements is approximately 5 HV0.1 (334 ± 8 HV0.1 for V powder, 329 ± 6 HV0.1 for U powder), and the obtained standard deviations are overlapping. The hardness of the solid samples is higher compared to the powder’s hardness. However, these are the typical values for the as-built IN718 produced by LPBF [47], as increased hardness is a result of characteristic IN718 microstructure caused by distinctive solidification conditions of the material in the LPBF process.

### 3.5. Powder Usability Rating

The presented research aims to evaluate the change of the properties of Inconel 718 powder, which is repeatedly used in the LPBF process, in order to optimize its reuse by introducing additional recycling steps between subsequent powder reuses (recycling cycles). To achieve this goal, a theoretical classification (powder usability rating) was adopted to quantify the correlations between the powders. A score of 0 is assigned to powders unsuitable for LPBF processing, and a score of 1 for powders that are suitable for LPBF. Four combinations of powder usability ratings were adopted:

- virgin and used = 1, overflow and spatter = 0 (Rating v1);
- virgin, used and overflow = 1, spatter = 0 (Rating v2);
- used and overflow = 1, virgin and spatter = 0 (Rating v3);
- used, overflow and spatter = 1, virgin = 0 (Rating v4).

**Figure 11:**
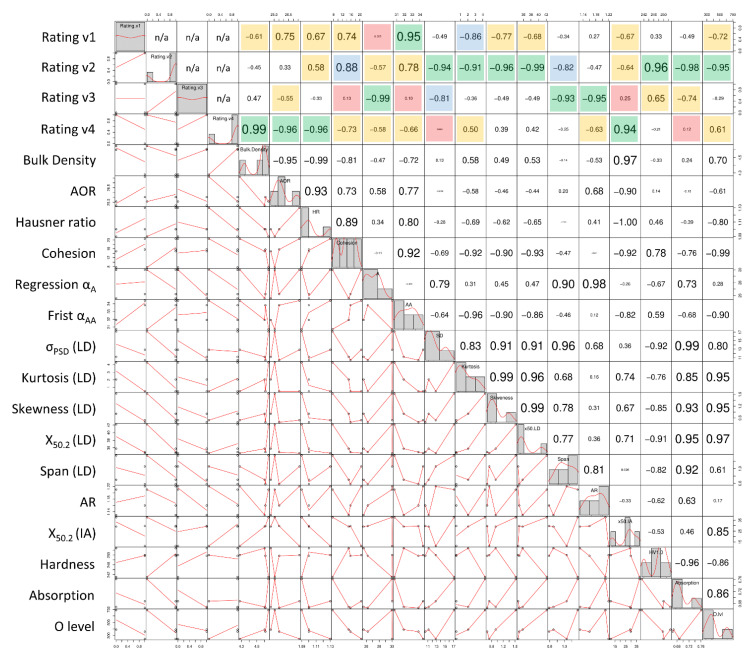
Matrix of dependence of the determined parameters of the powders in various processing states; green color—R ≥ |0.9|; blue color—|0.9|> R ≥ |0.8|; yellow color—|0.8|> R ≥ |0.5|; red color—|0.2| ≤ R.

For the purpose of the experiment, it was assumed that within a given combination of properties, the relationship between the individual results and powder usability rating, as well as between individual properties, is characterized by a linear correlation. The most representative properties of the Inconel 718 powder in all states, obtained during the studies, are summarized in Table 12. Based on Table 12, Pearson’s linear correlation coefficient R was determined for all the combinations of properties. A scatter-plot matrix, to better represent correlations, is shown in Figure 11.

If the R coefficient for the compared par of results is >|0.5|, it means that they are characterized by a strong correlation, while if R ≤ |0.5|, it is a sign of a poor correlation between them. R > 0 characterizes a proportional correlation, and R < 0 characterizes an inversely proportional correlation [62]. For the purpose of the experiment, a color scale was also adopted for individual R coefficients in order to better distinguish the differences between powder usability rating versions. The strongest dependencies were obtained for Rating v2, i.e., virgin, used and overflow = 1, spatter = 0. For almost all the measured parameters (13 out of 16 pairs), a strong correlation (R > |0.5|) was found between the measurement results and the usability rating. Only Bulk Density, Angle of Repose and Aspect Ratio showed weak or no correlation, mainly due to the properties of the virgin powder, which is characterized by a lower flowability when compared to the other powders. Rating v2 shows a very strong correlation (R ≥ |0.8|) for almost 60% of the combinations of properties. For the remaining three versions of usability ratings, high correlations between the rating and individual parameters can also be observed, but high R values occur less frequently than in Rating v2. The scatter-plot matrix also allowed for the evaluation of the correlations between powder parameters obtained in various measurement methods. Of all the measurement methods, a high number of large correlations (R ≤ |0.5|) was achieved for the laser absorption (in 11 out of 15 pairs). It was proven that by performing a laser absorption measurement, changes in all the three main groups of properties can be detected (i.e., morphology, flow and physico-chemical properties).

All the tested powder states are very similar to each other. Their properties hardly change during the successive reuse cycles in the LPBF process. The most degraded powder, not surprisingly, is spatter, but its degradation is lower than expected. The degree of degradation of the overflow powder is similar to that of the used powder. The U and O powder states are almost identical in terms of morphology, flow and physico-chemical properties. The virgin powder is a little different from the used and overflow powders, but as identified with the scatter-plot matrix analysis, the differences are small. The main difference between the V, U and O powders is due to repeated use. Repeated use causes the disappearance of the fine particles through the loss of the lightest powder particles in the powder feed system, sieves, filters and gas circulation systems of the LPBF machine and periphery devices.

The average density of the arrangement of parts on the LPBF platform is considered to be approximately 5–15%. Based on this value, it can be assumed that during a single powder reuse cycle, 10% of all the circulating powder is used to produce parts. Another 10% of the total powder batch is considered as waste, as it does not pass the screening. According to the research results, only a small portion of overflow powder is the spatter, which, due to the high PSD, increased oxygen level, higher flowability and higher laser absorption, may disturb the stability of the LPBF process. Therefore, it can be concluded that it is possible and recommended to apply a 2nd stage screening of overflow powder, which should be introduced as an additional step of powder recycling between reuses. After 2nd-stage screening, a mixing step of virgin, used and screed overflow powders can be introduced as the last stage of powder recycling between reuses. Mixing step proportions should be calculated based on real powder consumption. Laser absorption measurements can be introduced in the powder recycling process as a fast and efficient method of powder control. This powder recycling approach will allow the Inconel 718 utilization rate in the LPBF process to be significantly increased.

## 4. Conclusions

An evaluation of the metallic powder properties of Inconel 718 was performed in order to detect the level of degradation of powder in different states after repeated use in the Laser Powder Bed Fusion (LPBF) production cycle. The assessment included four types of powder states: V (virgin powder as supplied), U (used powder, after 20 LPBF cycles), O (overflow powder, rejected in the powder recycling process between reuses) and S (spatter powder, degraded powder particles due to powder/laser interactions during the LPBF process). The powders were analyzed for different properties that can be divided into three main categories: powder morphology, flow properties/flowability and physico-chemical properties.

The main findings are:

1. The most degraded powder was spatter, but its degradation was lower than expected. Degradation of the overflow powder was similar to the used powder. The used and overflow powders were almost identical in terms of morphology, flow, and physico-chemical properties. The fine particles in the virgin powder were the main reason for its differentiation (virgin compared to used and overflow powders). Moreover, it is mainly responsible for the lower flow properties of the virgin powder.
2. The properties of the Inconel 718 powder hardly changed during the successive reuse cycles in the LPBF process, and they showed a very high resistance to multiple reuses. Based on the obtained results, overflow powder, which is typically treated as waste, can be re-enabled for use in order to improve both the recycling performance of Inconel 718 powder, and the sustainability of the Laser Powder Bed Fusion process.

Detailed conclusions are:

3. All powder states were characterized by a spherical or spheroidal shape of particles. The shape of the powder, expressed by the AR and Circularity coefficients, did not degrade with reuse. The main differences in terms of the powders’ morphology were found in the PSD of the virgin and spatter powders when compared to the used and overflow powders. The virgin powder sample was characterized by the smallest PSD and had a large proportion of small particles (below the nominal PSD of 10-45 µm), which quantitatively constituted up to 50% of all the particles. The overflow and spatter powders showed a tendency for a right-skewed PSD, but for the overflow powder, the number of large particles was much less than in the spatter powder. The used and overflow powders are the most similar in terms of morphology and PSD.
4. It was found that the flow properties and flowability were significantly lower for the virgin powder when compared to the other powder states. The used, overflow and spatter powders were characterized by almost identical flow properties when static and semi-dynamic flow properties were compared. The spatter powder, however, was characterized by greater flowability in dynamic measurements (rotating drum analyzer). Nevertheless, irrespective of these differences, all the types of powders showed a sufficient level of flowability in terms of LPBF use.
5. All the powder particles, regardless of their degradation condition, were characterized by a low amount of gas pores and a low amount of LOF pores. The hardness and phase composition were also not affected due to the degree of degradation. The chemical composition of all the powder states is similar. The content ranges of H, N, Al, Cr and C, irrespective of the powder state, were not exceeded. The oxygen content in the spatter powder exceeded the limit value for IN718 according to ASTM B637 by 290 ppm. Moreover, aluminum oxide spots were found within some of the spatter particles. Laser absorption analysis showed great differences between spatter laser absorption (10 pp higher) when compared to the rest of the powders. The higher laser absorption of the spatter powder was found to be related to the thin oxidized layer on the surface of spatter powder particles. Laser absorption measurements were found to be a fast and efficient control method of Inconel 718 powder degradation in the course of its multiple LPBF reuse.

## Figures and Tables

**Table 1:** Chemical composition of the IN718 powder provided by SLM Solutions AG.

Powder	Ni	Cr	Fe	C	Ti	Al	Co	Mo	Nb	Mn	B	Cu	P	S	Si	H	N	O
ASTM B637 [wt.%]	50–55	17–21	Bal.	0.08	0.65–1.15	0.20–0.80	1.00	2.8–3.3	4.75–5.5	0.35	0.006	0.30	0.015	0.015	0.35	0.01	0.1	0.04
SLM Sol. [wt.%]	52.83	18.97	Bal.	0.04	0.96	0.60	0.03	2.99	5.05	0.03	0.003	0.02	0.004	<0.001	0.08	<0.01	<0.01	0.01

**Table 2:** Characterization of the IN718 powder samples in the different Laser Powder Bed Fusion (LPBF) processing states.

Powder	Description
Virgin (V)	Powder as delivered—taken from a sealed moisture-proof container with moisture-absorbing silica gel packets inside. The powder was not screened or otherwise conditioned before testing.
Used (U)	Powder used within the LPBF processes 20 times. After each use, the used powder was subjected to a standard, industrial recycling procedure, i.e., sieving with the use of a dedicated ultrasonic sieve (100 µm mesh) [24] in a protective atmosphere of argon (O_2_ level below 500 ppm). After screening, the powder was poured back into the residual tank operating under a shielding gas atmosphere.
Overflow (O)	Powder that accumulated in the waste container during successive screening cycles. The sieving device (PSM 100, SLM Solutions Group AG, Lübeck, Germany) is designed to quickly screen the powder in the production cycle (flow sieve). Before sampling, all the collected overflow powder was mixed in the entire volume with the use of a mechanical sieve shaker.
Spatter (S)	Spatter powder was collected from the area of the build chamber, close to the shielding gas outlet and upstream of the filter system. It is a mixture of used powder and one of 5 spatter types of powder particles (metallic jet spatter, solid spatter, agglomeration spatter, entrainment melting powder spatter and defect induced spatter [25]), which are generated during the LPBF process as a result of the instability of the liquid metal pool and dynamic laser movement [26]. Spatter powder was used as a control sample as a high degree of degradation was expected.

**Table 3:** Selected properties of metal powders in the context of PBF application and its measurement methods based on [9,13,14,22,28].

Section	Property	Measurement Method	Standards
Powder morphology	Particle-size Distribution (PSD)	Laser diffraction	ISO 13320-1, ASTM B822-17
		Image analysis SEM/LM	ISO 13322-1
	Particles’ morphology	Image analysis SEM/LM	n/a
Flow properties and flowability	Static	Bulk density	ISO 3923, ASTM B329
		Tap density	ISO 3953, ASTM B527, MPIF Standard 46
		Hall Flow Testing	ASTM B855/ASTM B213
		Angle of Repose (AOR)	Based on results of Hall Flow (ASTM B213)
	Dynamic	Cohesion (drum analyzer)	n/a
		Avalanche angle (drum analyzer)	n/a
Physico-chemical Properties	Chemical composition	XPS, AES, EDS, spectroscopy	Accredited measurements
	Microstructure	XRD	PN-EN 13925-1:2007
		Microscopic analysis	n/a
		Hardness	ISO 6507-1:2018, ASTM E92
	Other	Laser absorption	n/a

**Table 4:** Constant parameters of grain size measurements by laser diffraction.

Vacuum	Pressure	Feed Rate	Gap Width	Start/Stop
68 mbar	2 bar	60–70%	1.2 mm	C_opt_ ≥ 2.1%/C_opt_ ≤ 2.1%

**Table 5:** Constant parameters of the flow measurements using the GranuDrum material flow analyzer.

Rotation Speed [rpm]	Threshold	Images per Speed Level	Sampling [ms]
2, 5, 10, 20, 30, 40, 50, 60	101	30	1000

**Table 6:** Results of particle size distribution measurements of the IN718 powders in different states.

Parameter	Virgin (V)		Used (U)		Overflow (O)		Spatter (S)	
	Image	Diffraction	Image	Diffraction	Image	Diffraction	Image	Diffraction
Particles (n)	1167	-	916	-	983	-	1042	-
AR	1.22	-	1.16	-	1.13	-	1.21	-
Circularity	0.92	-	0.94	-	0.95	-	0.94	-
σ	-	12.85	-	10.40	-	11.18	-	17.14
Kurtosis	-	0.45	-	0.45	-	2.03	-	4.28
Skewness	-	0.72	-	0.65	-	0.94	-	1.63
x_10.2_ [µm]	4.48	19.62	14.51	22.63	14.29	23.21	15.45	25.91
x_50.2_ [µm]	11.25	34.60	29.64	34.88	29.13	35.78	37.71	42.15
x_90.2_ [µm]	24.63	55.23	54.26	50.30	56.85	53.45	75.05	75.63
Span	-	1.03	-	0.79	-	0.85	-	1.18

**Table 7:** Flow properties of the IN718 powders (±σ) in different states.

Flow Properties	Virgin (V)	Used (U)	Overflow (O)	Spatter (S)
Bulk density [g/cm^3^]	4.28 ± 0.01	4.61 ± 0.09	4.60 ± 0.03	4.65 ± 0.02
Tap density [g/cm^3^]	4.83 ± 0.04	5.06 ± 0.04	5.06 ± 0.02	5.04 ± 0.02
Hausner ratio	1.13 ± 0.01	1.10 ± 0.01	1.10 ± 0.01	1.09 ± 0.01
Angle of Repose [°]	27.63 ± 0.63	25.53 ± 0.52	24.66 ± 0.17	25.07 ± 0.07
Flow Time [s]	6.04 ± 0.16	4.88 ± 0.06	4.63 ± 0.13	5.93 ± 0.02

**Table 8:** Summary of flowability parameters for the IN718 powders in different processing states.

Parameter	Virgin (V)	Used (U)	Overflow (O)	Spatter (S)
Avg. cohesion index	20.6 ± 3.5	15.7 ± 3.9	14.9 ± 3.6	7.6 ± 2.7
Average α_AA_ [°]	43.9	41.4	40.2	41.6
Regression angle of α_AA_ curve [°]	30.03	26.23	25.57	29.99
First avalanche angle α_fAA_ [°]	34.4	33.8	31.9	30.7

**Table 9:** The hardness of the IN718 powders in all states (±σ).

Parameter	Virgin (V)	Used (U)	Overflow (O)	Spatter (S)
Hardness [HV0.1]	250.0 ± 13.42	249.7 ± 22.16	252.8 ± 23.63	241.1 ± 20.56
Avg. dia. of tested particles [µm]	46.6	58.5	82.2	86.5

**Table 10:** Chemical composition of critical elements/impurities for the IN718 powders in various states.

Element	Unit	Virgin (V)	Used (U)	Overflow (O)	Spatter (S)
Al	% wt.	0.54	0.55	0.54	0.54
Cr	% wt.	19.54	19.69	19.40	19.59
C	% wt.	0.041	0.042	0.042	0.043
N	% wt.	0.013	0.013	0.013	0.013
O	% wt.	0.025	0.039	0.036	0.069
H	% wt.	<0.0006	<0.0006	<0.0006	<0.0006

**Table 11:** Average laser absorption (±σ) of the IN718 powder in all states, measured for the full available wavelength spectrum (from 900 to 1100 nm).

Parameter	Virgin (V)	Used (U)	Overflow (O)	Statter (S)
Average laser absorption [%]	68.8 ± 0.7	66.6 ± 0.9	66.8 ± 0.8	76.1 ± 1.1

**Table 12:** Properties of the Inconel 718 powder (in all states) adopted in the scatter plot matrix model.

Property	Parameter	Virgin (V)	Used (U)	Overflow (O)	Spatter (S)
Evaluation	Usability rating v1 (0–1)	1	1	0	0
	Usability rating v2 (0–1)	1	1	1	0
	Usability rating v3 (0–1)	0	1	1	0
	Usability rating v4 (0–1)	0	1	1	1
Flow properties	Bulk density [g/cm^3^]	4.28	4.61	4.6	4.65
	Angle of Repose [°]	27.63	25.53	24.66	25.07
	Hausner ratio	1.13	1.10	1.10	1.09
Flowability	Avg. Cohesive index	20.6	15.7	14.9	7.6
	α_A_ [°]	30.03	26.23	25.57	29.99
	α_AA_ [°]	34.4	33.8	31.9	30.7
Particle size distribution	σ [µm]	12.85	10.40	11.18	17.14
	Kurtosis	0.45	0.45	2.03	4.28
	Skewness	0.72	0.65	0.94	1.63
	x_50,2 (LD)_ [µm]	34.60	34.88	35.78	42.15
	Span	1.03	0.79	0.85	1.18
Image analysis	Aspect ratio	1.22	1.16	1.13	1.21
	x_50,2 (IA)_ [µm]	11.25	29.64	29.13	37.71
Hardness	Vickers’s Hardness HV0.1	250.0	249.7	252.8	241.1
Laser absorption	Avg. laser absorption	0.688	0.666	0.669	0.761
Chemical composition	Oxygen level [ppm]	250	360	390	690

## Data Availability

The data supporting the findings of this study are available from the corresponding author on reasonable request.
